# Altering adsorbed proteins or cellular gene expression in bone-metastatic cancer cells affects *PTHrP* and *Gli2* without altering cell growth

**DOI:** 10.1016/j.dib.2015.07.003

**Published:** 2015-07-13

**Authors:** Jonathan M. Page, Alyssa R. Merkel, Nazanin S. Ruppender, Ruijing Guo, Ushashi C. Dadwal, Shellese Cannonier, Sandip Basu, Scott A. Guelcher, Julie A. Sterling

**Affiliations:** a Department of Chemical and Biomolecular Engineering, Vanderbilt University, Nashville, TN 37235, USA; b Department of Veterans Affairs, Tennessee Valley Healthcare System, Nashville, TN 37212, USA; c Center for Bone Biology, Vanderbilt University Medical Center, Nashville, TN 37232, USA; d Division of Clinical Pharmacology, Department of Medicine, Vanderbilt University Medical Center, Nashville, TN 37232, USA; e Department of Cancer Biology, Vanderbilt University Medical Center, Nashville, TN 37232, USA; f Agilent Technologies, Chandler, AZ 85226, USA; g Department of Biomedical Engineering, Vanderbilt University, Nashville, TN 37235, USA

## Abstract

The contents of this data in brief are related to the article titled “Matrix Rigidity Regulates the Transition of Tumor Cells to a Bone-Destructive Phenotype through Integrin β3 and TGF-β Receptor Type II”. In this DIB we will present our supplemental data investigating Integrin expression, attachment of cells to various adhesion molecules, and changes in gene expression in multiple cancer cell lines. Since the interactions of Integrins with adsorbed matrix proteins are thought to affect the ability of cancer cells to interact with their underlying substrates, we examined the expression of Integrin β1, β3, and β5 in response to matrix rigidity. We found that only Iβ3 increased with increasing substrate modulus. While it was shown that fibronectin greatly affects the expression of tumor-produced factors associated with bone destruction (parathyroid hormone-related protein, *PTHrP*, and *Gli2*), poly-l-lysine, vitronectin and type I collagen were also analyzed as potential matrix proteins. Each of the proteins was independently adsorbed on both rigid and compliant polyurethane films which were subsequently used to culture cancer cells. Poly-l-lysine, vitronectin and type I collagen all had negligible effects on *PTHrP* or *Gli2* expression, but fibronectin was shown to have a dose dependent effect. Finally, altering the expression of Iβ3 demonstrated that it is required for tumor cells to respond to the rigidity of the matrix, but does not affect other cell growth or viability. Together these data support the data presented in our manuscript to show that the rigidity of bone drives Integrinβ3/TGF-β crosstalk, leading to increased expression of *Gli2* and *PTHrP*.

Specifications table

**Table:** 

Subject area	Biology
More specific subject area	Cancer biology
Type of data	Figures
How data was acquired	ABI 7500 qPCR, western blot analysis, Image J software
Data format	Analyzed
Experimental factors	Adsorption of matrix proteins to PUR substrates, gene expression after silencing or over expressing
Experimental features	Gene expression of altered and unaltered bone-metastatic cancer cells was analyzed
Data source location	Nashville, TN USA
Data accessibility	The data is presented in this article and is related to [cite our article]

Value of the data

• Utilizing different matrix proteins shows that the bone destructive gene expression is highly specific to fibronectin.
• Manipulation of Integrin expression affects *PTHrP* and *Gli2* gene expression.
• Genetic manipulation of Integrin expression of MDA-MB-231 cells does not alter other metastatic pathways or the growth potential of the cell lines.

## 1 Data

### 1.1 Gene expression changes in response to rigidity

The bone-metastatic breast cancer cell line MDA-MB-231, bone-metastatic lung cancer cell line RWGT2, and the bone-metastatic prostate cancer cell line PC3 were used to test the effects of matrix rigidity on gene expression of the bone destructive genes, *PTHrP* and *Gli2*. Integrin β3 was either over expressed by an Iβ3 construct or inhibited in the MDA-MB-231 cell line by genetic inhibition using a shRNA construct or pharmacological inhibition with LM609 or Cilengitide [1].

**Fig. 1:**
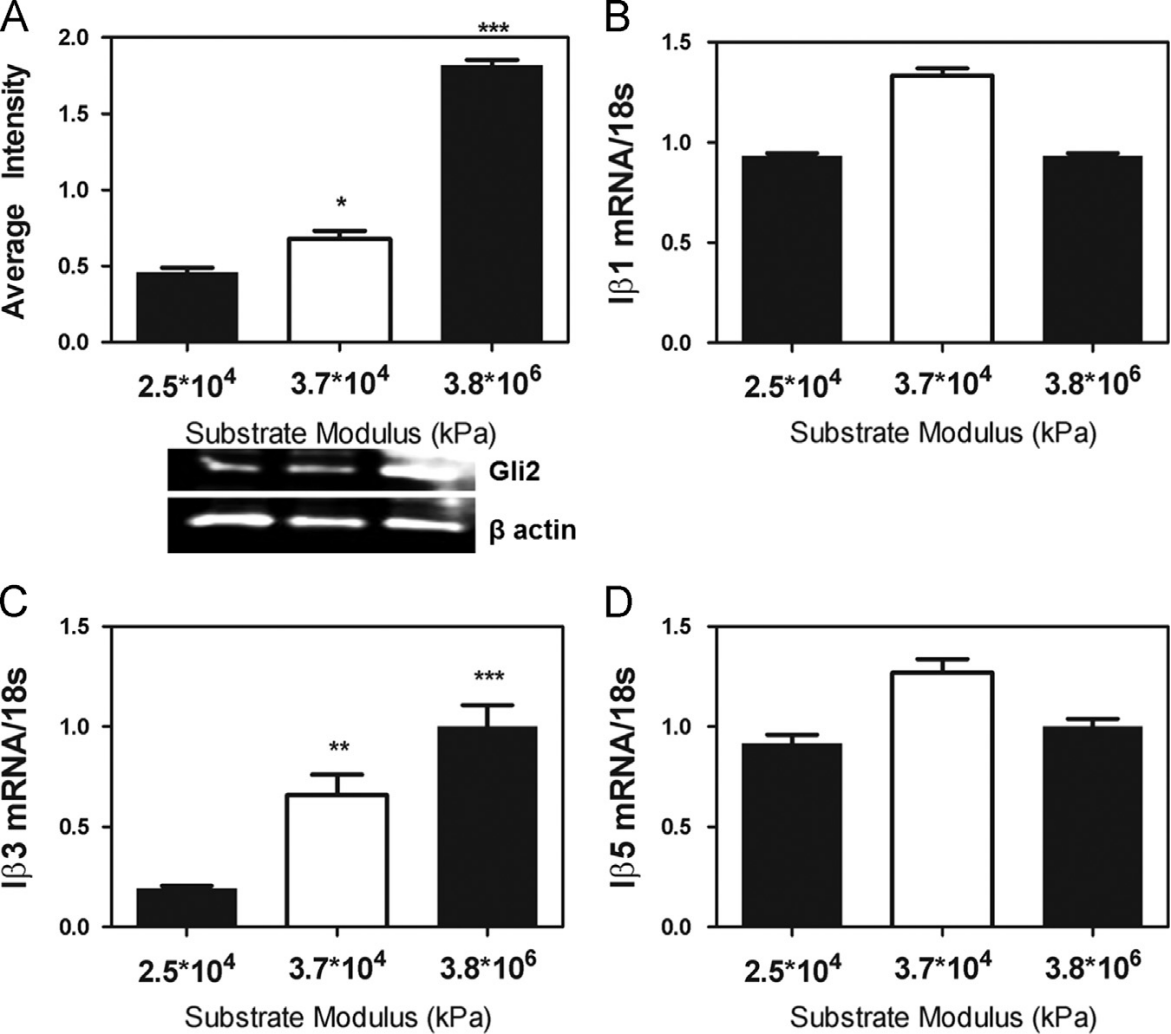
(A) *Gli2* protein expression increases with respect to substrate modulus. (B–D) Integrin β3 mRNA expression changes with increasing substrate modulus, while there is no change in Integin β1 and Integrin β5. (*p<0.05, **p<0.01, ***p<0.005). *N*=3 biological replicates. Data presented as fold change over 3.8×10^6^ kPa for each gene.

In addition to gene expression changes, *Gli2* protein increases with respect to matrix rigidity (Fig. 1A). While Integrin gene expression changes correlate with rigidity [2], in the bone-metastatic MDA-MB-231 cells, *Iβ1* and *Iβ5* do not respond to matrix rigidity while *Iβ3* increases with increasing substrate modulus (Fig. 1B D). Thus the cells are interacting with the matrix primarily through *Iβ3*.

### 1.2 Effects of adhesion Molecules on bone metastatic gene expression

**Fig. 2:**
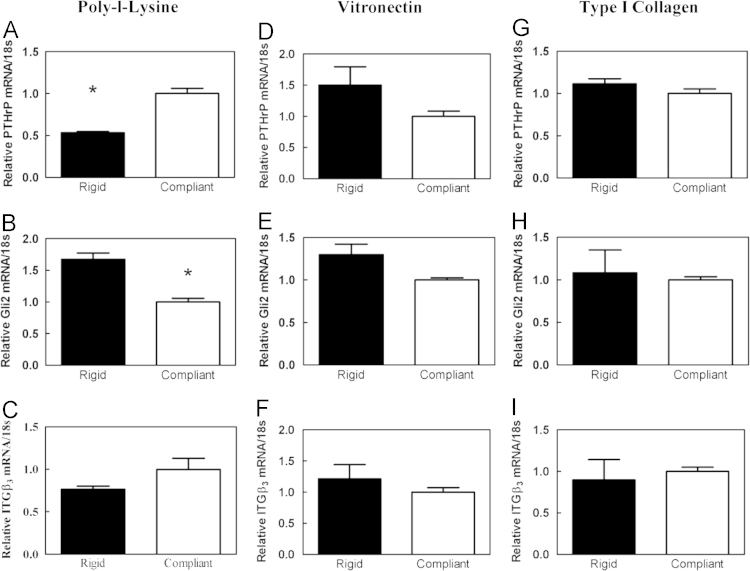
Expression of *PTHrP*, *Gli2*, and *Iβ3* by MDA-MB-231 cells on compliant and rigid PUR films treated with poly(L-lysine), vitronectin, or type I collagen. Gene expression was measured by qPCR. Data presented as fold change over compliant films.

Expression of *Iβ3*, *Gli2*, and *PTHrP* by MDA-MB-231 cells cultured on 2D compliant and rigid films and normalized to values measured for compliant films is shown in Fig. 2. With the exception of *PTHrP* expression on films treated with poly(L-lysine), no significant differences were observed between rigid and compliant films for poly(L-lysine), vitronectin, or type I collagen.

### 1.3 Inhibiting Iβ3 decreases *PTHrP* and *Gli2* gene expression

**Fig. 3:**
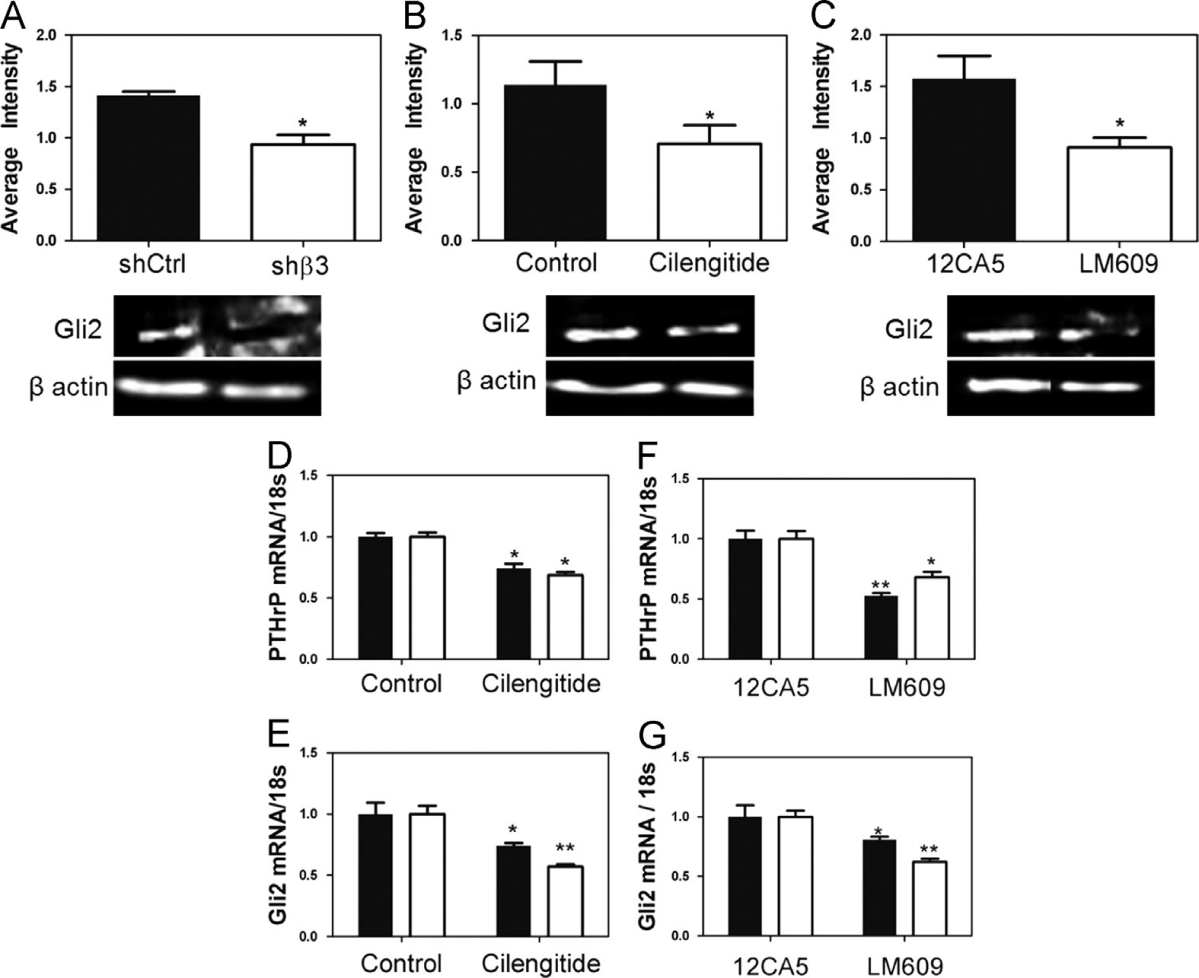
*Gli2* western blot for (A) shβ3 cells or (B–C) MDA-MB-231 cells treated with Cilentide or LM609. (D–G) *PTHrP* (D-E) or *Gli2* (F-G) mRNA expression for RWGT2 cells (black) or PC3 cells (white) treated with Cilengitide or LM609. (*p<0.05, **p<0.01). *N*=3 biological replicates. Data presented as fold change over untreated.

Genetic inhibition of *Iβ3* with shRNA in MDA-MB-231 cells decreased *Gli2* protein levels (Fig. 3A). Additionally, pharmacological inhibition with LM609 or Cilengitide decreased *Gli2* protein levels (Fig. 3B and C). Similar results were seen for *PTHrP* and *Gli2* when RWGT2 (black) or PC3 (white) cells were treated with LM609 or Cilengitide (Fig. 3D–G).

### 1.4 Molecular modulation of Iβ3 expression

**Fig. 4:**
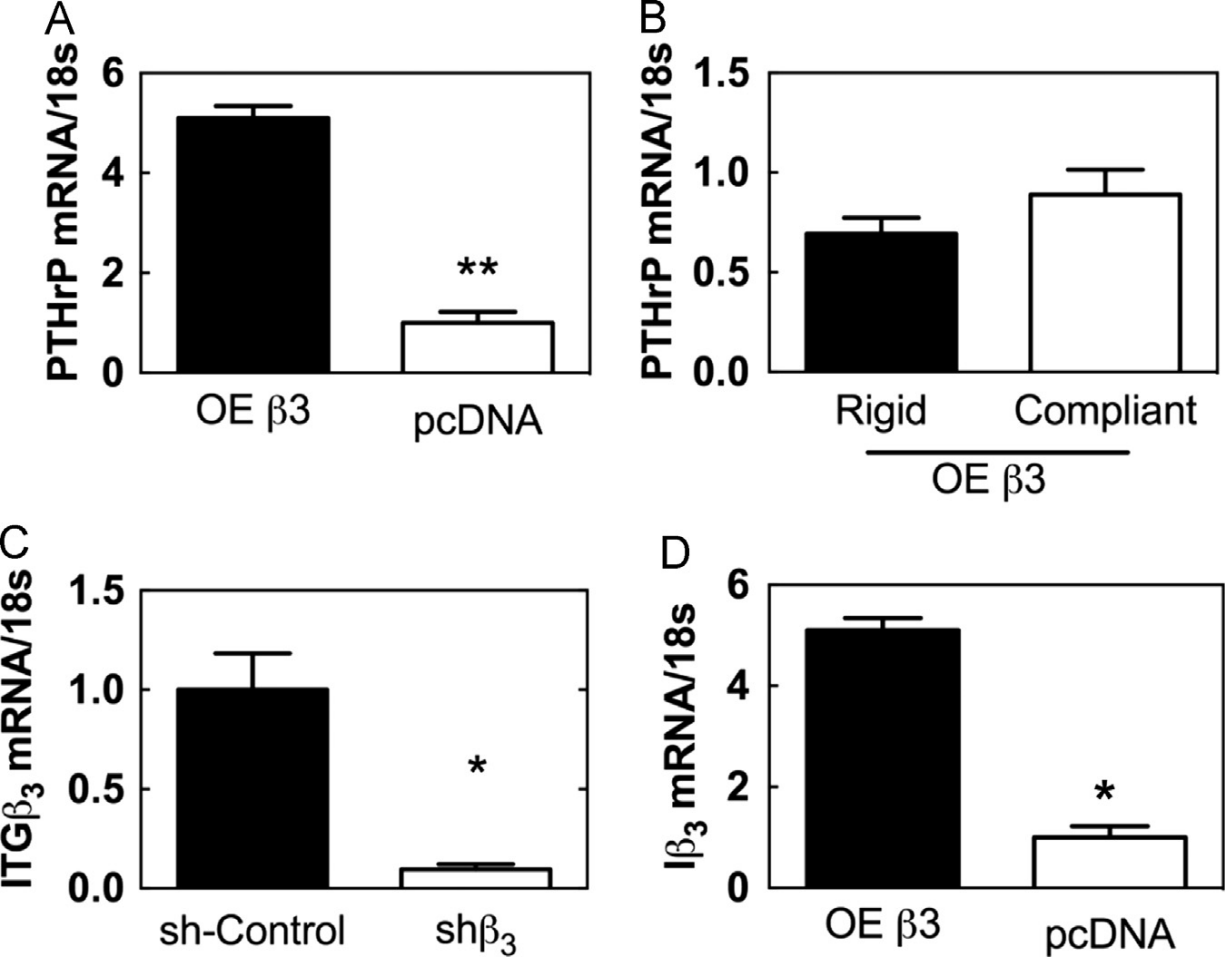
Expression of *PTHrP* and *Iβ3* in genetically modified MDA-MB-231 cells. (A) OE β3 cells over-express *Iβ3* compared to the mock-transfected control. (B) Effect of rigidity on expression of *PTHrP* by OE β3 cells. (C) shβ3 cells express significantly low *Iβ3* compared to the mock-transfected control. (D) OE β3 cells express significantly higher *Iβ3* compared to the mock-transfected control. (^⁎^*p*<0.05, ^⁎^^⁎^*p*<0.01). *N*=3 biological replicates. Data presented as fold change over untreated or compliant.

OE β3 cells showed increased *PTHrP* expression compared to mock-transfected control cells (Fig. 4A). Additionally, when cultured on rigid and compliant PUR films, the OE β3 cells showed no statistical difference in *PTHrP* gene expression (Fig. 4B). Thus, the OE β3 and shβ3 cells were utilized as model cell systems with high and low Integrin expression, respectively (Figs. 4C and D).

### 1.5 Effects of matrix rigidity on *TGF-β RII* expression

**Fig. 5:**
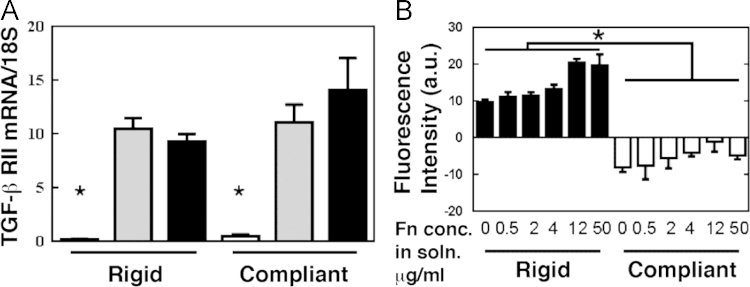
(A) Effects of matrix rigidity on TGF-β RII expression by MCF-7 (white), MDA-MB-231 (gray) and RWGT2 (black) cells. (B) Effects of Fn concentration on the FRET signal for MDA-MB-231 cells.

Expression of *TGF-β RII* by MCF-7 (negative control), MDA-MB-231, and RWGT2 cells was measured by qPCR on rigid and compliant substrates. As anticipated, expression of *TGF-β RII* was significantly lower in MCF-7 cells compared to MDA-MB-231 and RWGT2 cells. There were no significant differences in expression as a function of matrix rigidity for any of the three cell types (Fig. 5A).

### 1.6 Effects of Fn concentration on physical interactions between *Iβ3* and *TGF-βRII*

The effects of Fn concentration on the FRET signal in MDA-MB-231 cells was measured for Fn concentrations ranging from 0–50 μg/ml. The FRET signal was significantly higher on rigid substrates at all Fn concentrations (Fig. 5B).

### 1.7 Exogenous TGF-β stimulation of *PTHrP* and *Gli2* is *Iβ3*-dependent

**Fig. 6:**
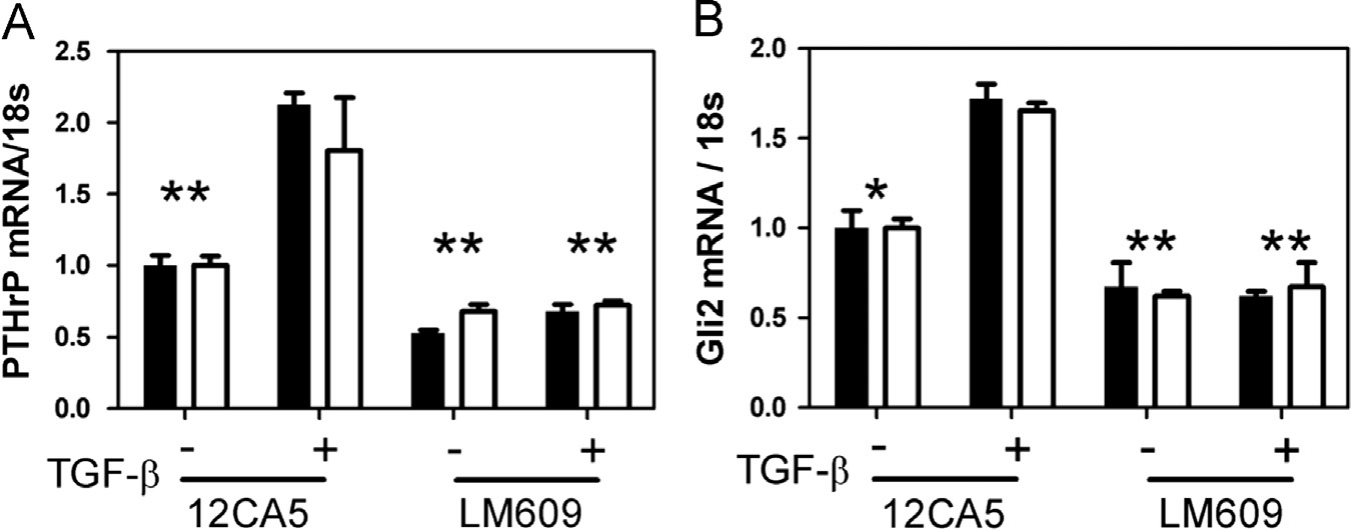
(A) *PTHrP* and (B) *Gli2* mRNA expression for RWGT2 (black) or PC3 (white) cells treated with LM609 and given exogenous TGFβ. (*p<0.05, **p<0.01, ***p<0.005). *N*=3 biological replicates. Data presented as fold change over untreated.

To investigate the role of TGF-β signaling in *Iβ3* regulation of *PTHrP* and *Gli2*, RWGT2 or PC3 cells were treated with the Integrin inhibitory antibody LM609 and given exogenous TGF-β. *PTHrP* and *Gli2* were analyzed by qPCR. TGF-β stimulates *PTHrP* and *Gli2* mRNA expression in RWGT2 (black) or PC3 (white) cells, but is unable to stimulate expression when *Iβ3* is inhibited with LM609 (Fig. 6A and B) suggesting that both TGF-β and *Iβ3* are required for regulating *PTHrP* and *Gli2*.

### 1.8 Effects of silencing *Iβ3* on growth of and bone metastatic gene expression by MDA-MB-231 tumor cells

**Fig. 7:**
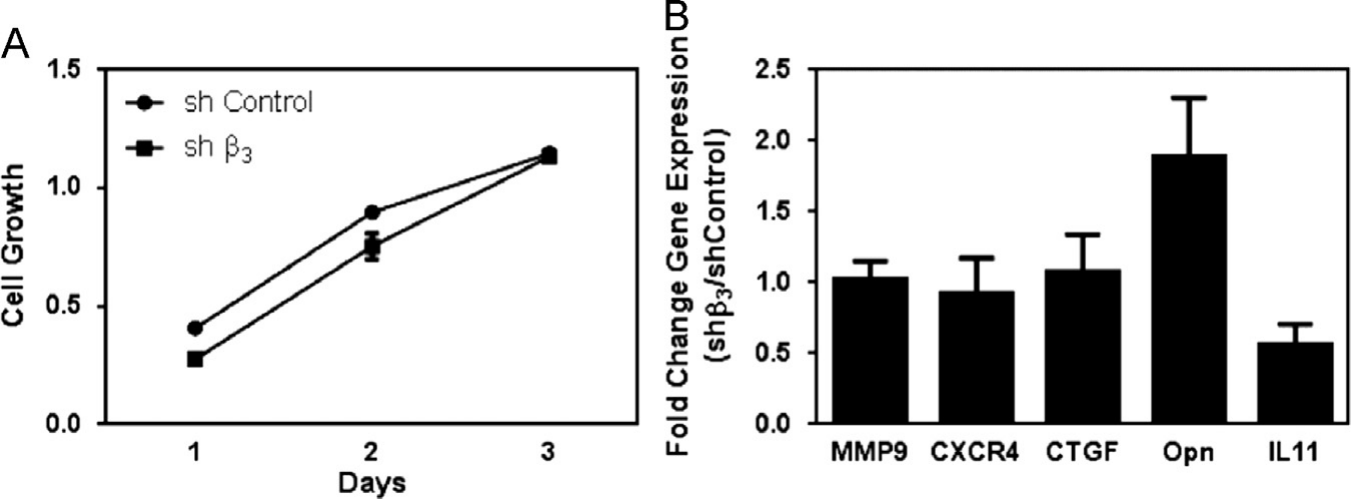
Effects of silencing *Iβ3* on tumor cell growth and bone metastatic gene expression. (A) Cell growth. (B) Bone metastatic gene expression.

As shown in Fig. 7A, shβ3 cells exhibited a growth rate similar to that of the mock-transfected control. With the exception of Osteopontin (Opn), expression of bone metastatic genes by shβ3 cells was similar to that by mock-transfected control cells (Fig. 7B).

## 2 Experimental design, materials, and methods

### 2.1 Western blot analysis

Cells were harvested 24 h after seeding on PUR substrates in a radioimmunoprecipitation buffer containing a cocktail of protease and phosphatase inhibitors (Pierce). Equal protein concentrations were prepared for loading with NuPAGE sample buffer (Life Technologies) and separated on a 10% SDS-PAGE gel (BioRad). Proteins were transferred to a PVDF membrane and blocked with 5% BSA in TBS containing 0.1% Tween-20 for 1 h at room temperature, followed by incubation with anti-*Gli2* antibody (1:1000, SantaCruz) overnight at 4 °C. After washing, membranes were blotted with anti-goat IgG (1:2000, SantaCruz), and bands were detected by enhanced chemiluminescence using an In-Vivo MS FX Pro (Bruker). Membranes were then stripped and reprobed using an antibody for β-actin (1:5000, Sigma) as a loading control. Analysis was performed using Image J software.

### 2.2 Quantitative real-time PCR

To measure changes in gene expression, mRNA reverse transcription was carried out using the qScript cDNA synthesis kit (Quanta, VWR) per manufacturer׳s instructions. Briefly, cells were harvested with trypsin after 24 h in culture and total RNA was extracted using the RNeasy Mini Kit (Qiagen). The qScript cDNA supermix was used to synthesize cDNA using 1 µg total RNA. The expression of *PTHrP*, *Gli2*, *Iβ1, Iβ3, Iβ5,* and *TGF-β RII* was measured in triplicate by quantitative qRT-PCR using validated TaqMan primers with the 7500 Real-Time PCR System (Applied Biosciences) using the following cycling conditions: 95 °C for 15 s and 60 °C for 1 min, preceded by an initial incubation period of 95 °C for 10 min. Quantification was performed using the absolute quantitative for human cells method using 18S as an internal control.

### 2.3 Adsorption of adhesion molecules

In addition to fibronection (Fn), compliant and rigid PUR films were incubated with a 4 µg/mL solution of vitronectin, type I collagen, or poly(L-lysine) in PBS overnight at 4 °C to mediate cell adhesion. MDA-MB-231 cells were cultured on the films for 48 h. Expression of *Iβ3*, *Gli2*, and *PTHrP* was measured by qPCR relative to the control 18S.
